# Stereotactic Body Radiation Therapy for Intrahepatic Graft Recurrences of Hepatocellular Carcinoma After Liver Transplantation: A Report of Two Cases

**DOI:** 10.7759/cureus.85536

**Published:** 2025-06-07

**Authors:** Alexander Piening, Alyssa Capizzi, Jeevin Shahi

**Affiliations:** 1 Department of Radiation Oncology, Saint Louis University School of Medicine, St. Louis, USA; 2 Department of Radiation Oncology, SSM Health Saint Louis University Hospital, St. Louis, USA

**Keywords:** case study, hepatocellular carcinoma recurrence, liver transplantation, retrospective analysis, stereotactic body radiation therapy

## Abstract

The management of intrahepatic recurrences after orthotopic liver transplantation (OLT) for hepatocellular carcinoma (HCC) is challenging. Graft hepatectomy may not be feasible, and systemic therapy (ST) options are limited due to the increased risk of graft rejection with immunotherapy use. Stereotactic body radiation therapy (SBRT) is a safe and effective locoregional therapy (LRT) option for HCC, although there is a paucity of data in the post-OLT setting. Here, we present two cases of post-OLT intrahepatic HCC recurrences treated with SBRT.

Two patients who received SBRT for post-OLT intrahepatic recurrences were identified from a single academic institution with a high-volume liver transplant program. Prior to treatment, cases were reviewed at a multidisciplinary liver tumor board. Baseline and treatment characteristics, as well as clinical outcomes such as local control (LC), progression-free survival (PFS), and adverse events (AEs), were retrospectively evaluated from the date of SBRT completion to last follow-up. Linear accelerator-based liver SBRT was planned using four-dimensional computed tomography (4DCT) simulation, abdominal compression, and volumetric modulated arc therapy. Institutional planning objectives were followed to achieve PTV95%>99% and to limit liver D_mean_ <18 Gy and D_700cc _<15 Gy.

Patient 1 (P1) and patient 2 (P2) underwent OLT for hepatitis C virus-associated HCC. The SBRT indication was for synchronous intrahepatic and oligometastatic progression without prior LRT (P1) and metachronous intrahepatic progression after five courses of non-SBRT LRT (P2). Time from OLT to SBRT was 6.7 years (P1) and 23.2 years (P2). Post-SBRT follow-up time was 20.9 months (P1) and 18.1 months (P2). Liver SBRT dose was 35 Gy in five fractions. P1 received synchronous SBRT to oligometastases involving the right adrenal gland and right lung. Intrahepatic PTV volumes were 230 cc (P1) and 114 cc (P2). For all treated lesions (n=5), LC was 100%. PFS was 9.7 months (P1) and 10.4 months (P2), with out-of-field intrahepatic progression being the only site of progression in both patients. No SBRT-related AEs were identified, and Child Pugh A status was maintained over the follow-up period. Neither patient received ST prior to SBRT; however, due to progression, P1 was started on first-line levanetinib 13 months after SBRT completion.

SBRT was effective and well tolerated in the treatment of two patients with intrahepatic HCC recurrences following OLT for HCC. Individualized treatment approaches incorporating SBRT for intrahepatic and extrahepatic recurrences should be considered in this population. Further analyses are warranted to compare the safety and efficacy of SBRT to other LRT options and in combination with ST in this patient population.

## Introduction

Hepatocellular carcinoma (HCC) remains a significant health burden worldwide as incidence and mortality rates continue to climb despite continued research, advancements in systemic therapy (ST) strategies, and ongoing hepatitis vaccination campaigns [1]. While there are a variety of locoregional treatment (LRT) options for early-stage HCC, such as surgical resection, radiofrequency ablation (RFA), transcatheter arterial chemoembolization (TACE), Y90 transarterial radioembolization (TARE), stereotactic body radiation therapy (SBRT), and particle beam therapy, the preferred curative-intent modality is orthotopic liver transplantation (OLT) [2,3].

Following OLT, five-year survival rates range from 70-80%; however, approximately one in six patients will ultimately develop recurrent HCC, which has a poor median survival of 12.2 months [4,5]. Of transplant patients who develop recurrent HCC, approximately one-third have a solitary intrahepatic graft recurrence that may be salvageable with resection or LRT [5-7]. For patients with extrahepatic recurrences or disseminated disease, the mainstay of treatment consists of systemic therapy (ST) with or without additional local salvage. The best LRT option depends upon multidisciplinary evaluation, surgical candidacy, intra- and extrahepatic tumor burden, as well as anatomical relationships to vasculature and adjacent organs-at-risk (OARs), such as stomach, bowel, and bile duct [6]. SBRT may offer several advantages in this setting compared to surgical resection or other LRT options, as it is an ablative, non-invasive external beam technique with steep dose fall-off, minimizing damage to the uninvolved liver graft and surrounding healthy tissues.

While SBRT is established as an LRT option for HCC due to its efficacy and safety profile, there is a paucity of data in the management of post-OLT intrahepatic disease due to decreased incidence compared to primary HCC and the relative novelty of SBRT as a treatment option compared to resection and historical LRT options. A study by Au et al. analyzed six patients with intrahepatic graft recurrences treated with SBRT and demonstrated impressive local control (LC) without impairment of graft function and minimal treatment-related toxicities [7]. Given the limited experiences in this clinical setting, here we present two patients who received SBRT for the management of post-OLT intrahepatic recurrences of HCC.

## Case presentation

Materials and methods

Two patients who received SBRT for post-OLT intrahepatic recurrences were identified from our academic institution.

Patient 1 was diagnosed with recurrence via magnetic resonance imaging (MRI), which revealed interval growth of a previously stable right lower lung (RLL) nodule (1.7 x 0.8 cm). Follow-up positron emission tomography/computed tomography (PET/CT) showed mild uptake in this lesion with a standardized uptake value maximum (SUVm) of 2.0 and a right adrenal gland nodule (1.1 cm) with an SUVm of 4.3. RLL biopsy failed due to tumor motion, and right adrenal gland biopsy showed fibrous tissue. Follow-up CT four months later showed further growth of the RLL nodule to 2.5 cm, a new 7 mm RLL nodule, and an increase in size of the adrenal gland nodule to 1.7 cm. The patient was then lost to follow-up for 13 months, at which point MRI showed growth of the patient’s adrenal mass to 4.2 x 6.5 cm and a new enhancing 4.2 x 5.5 cm exophytic mass in the right hepatic lobe. At this time, Patient 1 had an alpha-fetoprotein (AFP_ of 31.4, and on multidisciplinary imaging review, all these sites were consistent with metastatic HCC.

Patient 2 was diagnosed with intrahepatic recurrence via increasing AFP levels and ultrasound monitoring demonstrating two hypoechoic lesions. Ultrasound was subsequently confirmed via MRI, as the imaging characteristics were consistent with HCC. At the time of diagnosis via imaging, Patient 2 had an AFP of 317.2.

Both patients denied any abdominal pain, nausea, vomiting, or other GI complaints. Physical exam was negative for right upper quadrant tenderness, jaundice, or masses. Both patients were reviewed at a multidisciplinary liver tumor board comprising specialists in radiation oncology, hematology and oncology, gastroenterology, hepatology, radiology, and pathology. After discussion at the tumor board, the final management plan was to proceed to SBRT to the involved sites of disease and consider ST for additional progression, if required.

Patient 1 presented for treatment with SBRT in July 2021, and Patient 2 presented for treatment with SBRT in March 2022. The inclusion of both cases was consecutive. Multiple imaging modalities, including CT and MRI, were evaluated to assess tumor characteristics, location, and relationship to surrounding OARs. Patient information, treatment characteristics, and outcomes such as LC, progression-free survival (PFS), overall survival (OS), and adverse events (AEs) were retrospectively evaluated from the time of SBRT completion. Patient-specific information and treatment characteristics are outlined in Table 1.

**Table 1:** Patient-specific information and treatment characteristics ECOG PS: Eastern Cooperative Oncology Group performance status; HCC: hepatocellular carcinoma; SBRT: stereotactic body radiation therapy; LRT: locoregional therapy; TACE: transcatheter arterial chemoembolization; PTV: planning target volume; Gy: Gray; PFS: progression-free survival; OS: overall survival. PTV refers to a volume for treatment that takes into account uncertainties in treatment delivery, such as patient position, organ motion, and beam alignment. D700cc refers to the dose that 700 cc of liver was exposed to.

Characteristics	Patient 1	Patient 2
Age	70 years	75 years
Sex	Female	Male
ECOG PS	1	1
Child-Pugh Class	A	A
HCC etiology	Hepatitis C virus	Hepatitis C virus
SBRT indication	Intrahepatic and oligometastatic progression	Intrahepatic progression
Time from OLT to SBRT	6.7 years	23.2 years
LRT to OLT prior to SBRT	No	Microwave ablation x2, TACE x1
Transplant rejection regimen (continued during SBRT)	Tacrolimus 5 mg daily	Sirolimus 1 mg every other day
Liver SBRT dose	35 Gy in 5 fractions	35 Gy in 5 fractions
Number of liver isocenters	1	1
Other sites treated with SBRT	Yes: right adrenal gland, right lung x2	No
Concurrent systemic therapy	No	No
Intrahepatic PTV volume	230 cc	114 cc
Plan D_max_	153% (composite)	117%
Liver D_mean_	16.6 Gy	12.5 Gy
Liver D_700cc_	14.1 Gy	10.6 Gy

Patient 1 and Patient 2 underwent OLT for hepatitis C virus-associated HCC. SBRT indication was for synchronous intrahepatic and oligometastatic progression without prior LRT (Patient 1) and metachronous intrahepatic progression after five courses of non-SBRT LRT (Patient 2). Time from OLT to SBRT was 6.7 years (Patient 1) and 23.2 years (Patient 2). The liver SBRT dose selected was 35 Gy in five fractions and was based on physician preference to deliver a modest SBRT dose without OAR compromise, as well as established protocols for liver SBRT [8]. Patient 1 also received synchronous SBRT (35 Gy in five fractions) to a right adrenal gland metastasis and two right lung oligometastases. Intrahepatic planning target volume (PTV) was 230 cc (Patient 1) and 114 cc (Patient 2).

**Figure 1:**
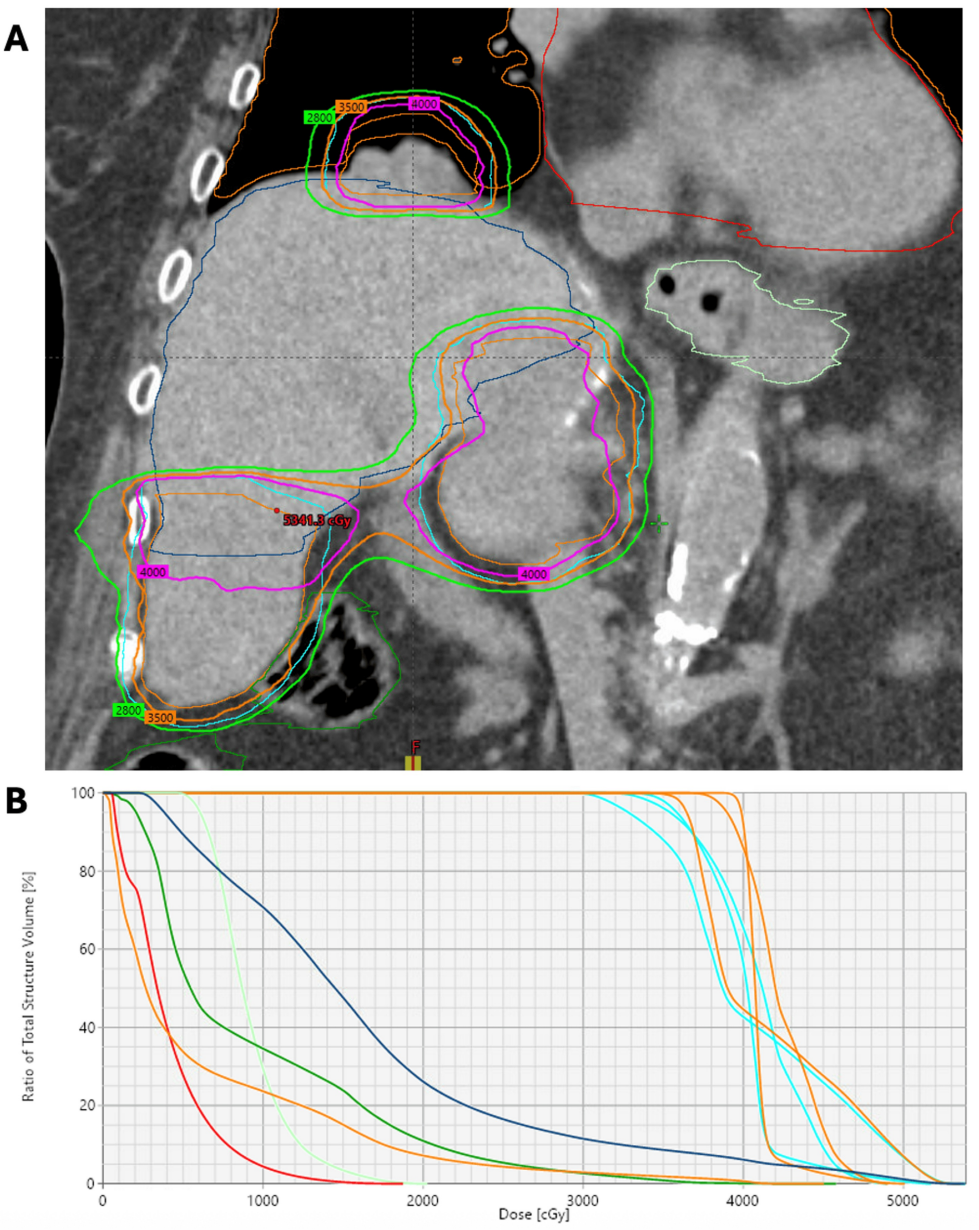
SBRT dose distribution in Patient 1 (A) Coronal view of the composite SBRT isodose distribution for Patient 1. She received synchronous, multi-isocentric SBRT to a dose of 35 Gy in five fractions to three separately involved sites (segment 8 liver graft, right adrenal gland, and right lung). Composite isodose levels shown are 40 Gy (pink), 35 Gy (orange), and 28 Gy (green). The three internal target volumes and planning target volumes are shown in orange and cyan, respectively. Adjacent organs-at-risk included the large bowel (dark green), stomach (light green), lungs (orange), heart (red), and liver (dark blue). (B) The accompanying composite dose volume histogram is also shown. SBRT: stereotactic body radiation therapy

**Figure 2:**
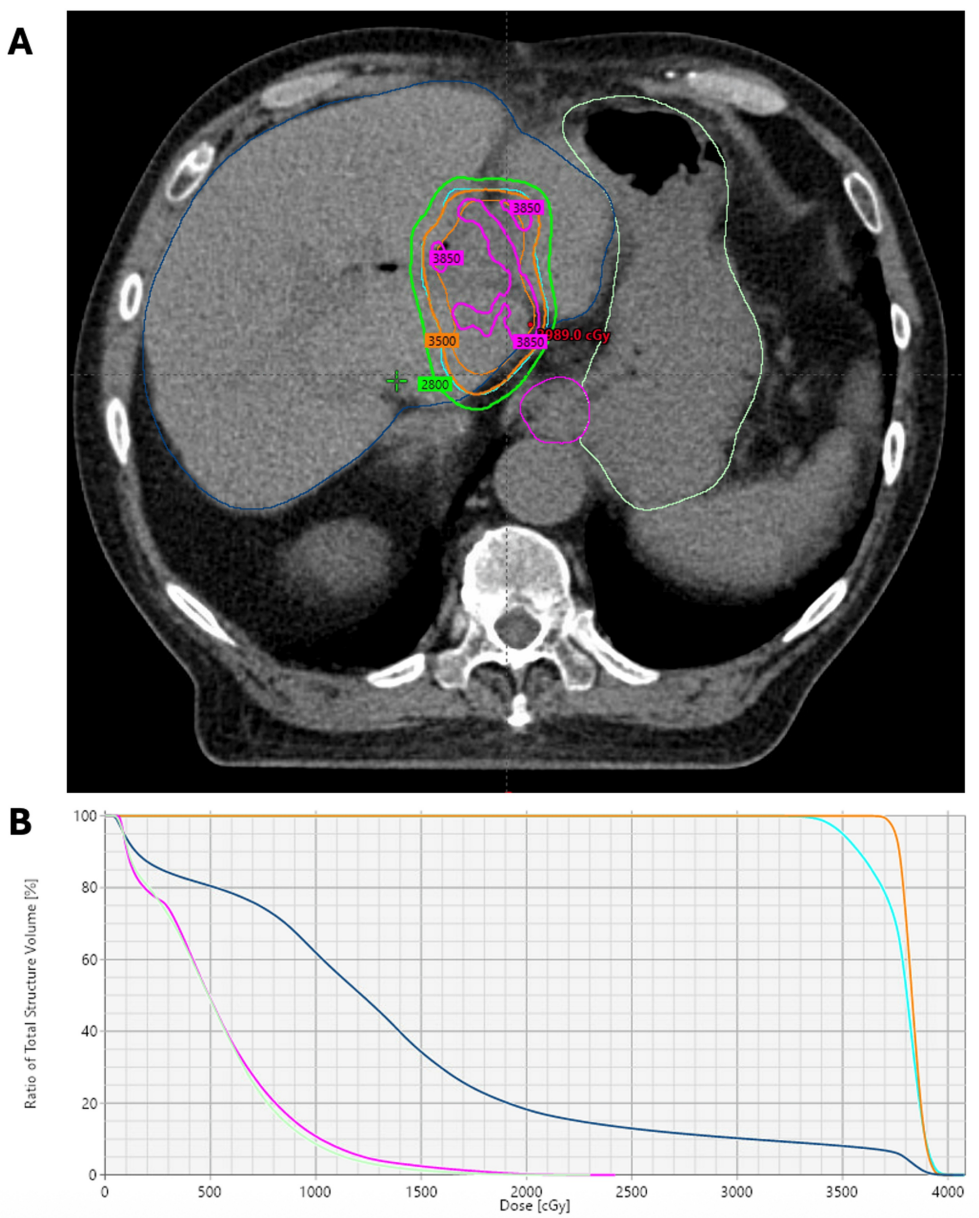
SBRT dose distribution in Patient 2 (A) Axial view of the SBRT isodose distribution for Patient 2. He received SBRT to a dose of 35 Gy in five fractions to an intrahepatic graft recurrence in segments 1/4A). The isodose levels shown are 38.5 Gy (pink), 35 Gy (orange), and 28 Gy (green). The internal target volume and planning target volume are shown in orange and cyan, respectively. Adjacent organs-at-risk included the stomach (light green), esophagus (pink), and liver (dark blue). (B) The accompanying composite dose volume histogram is also shown. SBRT: stereotactic body radiation therapy

Linear accelerator-based liver SBRT was planned using four-dimensional (4D)-CT simulation, abdominal compression with a pneumatic abdominal compression belt (Body Pro-Lok ONE™; CQ Medical, Avondale, Pennsylvania, United States), and volumetric modulated arc therapy (VMAT) with two coplanar arcs. The patients were treated using a TrueBeam linear accelerator (Varian Medical Systems Inc., Palo Alto, California, United States) and 10 MV photons using a flattening filter-free approach. Dose constraints utilized were according to established SBRT guidelines [9]. Treatments were administered free-breathing and without respiratory gating or tumor tracking. Particle beam therapy and/or spacer insertion were not recommended due to the lack of availability of these technologies at our center. Institutional planning objectives were followed to achieve PTV95%>99% and to limit liver Dmean <18 Gy and D700cc <15 Gy. SBRT dose distributions for Patient 1 and Patient 2 are demonstrated in Figure 1 and Figure 2, respectively.

Post-SBRT follow-up time was 20.9 months (Patient 1) and 18.1 months (Patient 2). Following SBRT, patients were monitored with regularly scheduled surveillance imaging to assess LC and with clinic visits to assess potential AEs. CT chest, abdomen, and pelvis without contrast and MRI with and without contrast were obtained every 3 months following SBRT for surveillance.

Results

In both patients, no SBRT-related AEs were identified, and Child-Pugh A scores were maintained throughout the follow-up period. Specifically, neither patient exhibited significant fatigue, skin changes, nausea, vomiting, diarrhea, loss of appetite, or bleeding. For all treated lesions in both patients (n=5), LC was 100%. Patient outcomes, including local progression, PFS, and OS following SBRT, are outlined in Table 2.

**Table 2:** Patient outcomes following SBRT treatment SBRT: stereotactic body radiation therapy; PFS: progression-free survival; OS: overall survival; HCC: hepatocellular carcinoma

Outcomes	Patient 1	Patient 2
Post-SBRT local progression	No	No
Post-SBRT PFS	9.7 months	10.4 months
Post-SBRT OS	25.8 months	25.6 months
De novo out-of-field progression	Yes	Yes
Cause of death	Progressive HCC	Progressive HCC

Post-SBRT PFS was 9.7 months (Patient 1) and 10.4 months (Patient 2), with out-of-field intrahepatic progression being the only site of progression in both patients. Post-SBRT OS was 25.8 months (Patient 1) and 25.6 months (Patient 2). Neither patient received ST prior to SBRT; however, due to progression, Patient 1 was started on first-line leucovorin 13 months after SBRT completion. Neither patient received immunotherapy due to concerns regarding graft rejection.

## Discussion

In two patients with post-OLT HCC recurrences treated with SBRT, we found that SBRT achieved 100% LC for all treated lesions, was associated with a favorable post-treatment OS, and did not cause any apparent treatment-related AEs. Out-of-field progression occurred in both patients and was not unexpected given the aggressive natural history of recurrences following OLT. The optimal management of HCC graft recurrences is complex, owing to several clinical factors including performance status, liver function, immunosuppression, concern regarding graft rejection, tumor staging, resectability, and intrahepatic and extrahepatic disease burden [10]. In general, the strategy for treatment is based on multidisciplinary input and can be simplified based on the extent of recurrent disease as determined by restaging imaging: intrahepatic (confined to the liver graft) and/or extrahepatic, which can be further separated into oligometastatic (limited to ≤ 5 metastatic sites) or widely disseminated (>5 metastatic sites) disease [10,11].

In the setting of confined intrahepatic disease, it may be feasible to proceed with resection or other LRT options depending on tumor burden, volume of uninvolved liver, graft function, and patient tolerability [10]. When surgery is not a viable option, established definitive LRT modalities such as SBRT, RFA, TACE, and Y90 may be used for the salvage of graft recurrences post-OLT [3]. While RFA has demonstrated comparable long-term outcomes to resection in the management of post-OLT HCC recurrences, the effectiveness of RFA may be limited by tumor size and the heat-sink effect to nearby organs or vasculature [12]. In fact, a recent randomized trial reported a significant LC advantage of SBRT over RFA in patients with recurrent HCC, demonstrating that SBRT may actually provide superior intrahepatic control [13].

Similarly, altered post-transplant anatomy can pose challenges to the administration of TACE/TARE, and there remains concern regarding potential toxicity to graft vasculature with these techniques. Compared to other LRT modalities, SBRT may offer several potential advantages, including non-invasive treatment, lack of anatomical limitations to dose delivery, the ability to treat multiple intrahepatic and extrahepatic sites, and the incorporation of physician-defined margins for geometric uncertainties and microscopic disease within the grafted liver. SBRT, however, is not without the risk of potential adverse events such as radiation-induced liver disease (RILD) and damage to critical adjacent OARs (such as stomach, bowel, bile ducts, and blood vessels). In the non-transplant setting, liver SBRT has an acceptable toxicity profile with grade 3 or greater AEs typically reported in <10% of patients, although these experiences may not directly translate to the post-transplant setting [14]. Risks related to SBRT may be mitigated with careful attention to post-transplant grafting and anatomical variations, accurate definition of treatment margins, and the utilization of advanced radiation delivery techniques (such as VMAT, respiratory motion management, intrafraction monitoring, and/or proton therapy) to reduce doses to nearby OARs. We opted to use modest SBRT doses (35 Gy in five fractions delivered every other day) to potentially reduce the risk of any damage to the liver graft, vasculature, or adjuvant OARs. In both our study and the study by Au et al. [7], there was no evidence of graft toxicity and minimal reported SBRT-related AEs [7]. Given the small sample size of these experiences, however, the utilization of post-OLT liver SBRT should be undertaken with caution and only by radiation oncologists with expertise in the planning and delivery of these treatments. We must also emphasize the importance of multidisciplinary collaboration with liver transplant specialists.

While the use of liver SBRT for limited intrahepatic HCC is established, it is unclear if there is any substantial benefit in patients with extrahepatic (metastatic) disease. In the post-transplant setting, the effect of local therapy may be less impactful on PFS, as even patients with graft-confined recurrences can be argued to have metastases disseminating from the native liver [10]. There is the possibility that a secondary graft HCC may develop in the setting of prolonged immunosuppression, which may explain the long disease-free interval from OLT to diagnosis (6.7 years and 23.2 years) in our two patients. Since post-OLT HCC patients cannot receive immunotherapy due to the fear of graft rejection, potentiating the effectiveness of non-immunotherapy ST options, such as tyrosine kinase inhibitors (TKIs), by combining treatment with SBRT should be investigated. A retrospective review of the National Cancer Database suggested that patients with stage IV HCC who received SBRT had improved OS compared to those who received ST alone [15]. Given these findings, there may be a clinical benefit in treating metastatic sites of HCC with SBRT, as has been demonstrated in the oligometastatic setting in other patient populations [11].

The results of Radiation Therapy Oncology Group (RTOG) 1112 support this potential paradigm, as this phase 3 trial demonstrated that combined treatment of locally advanced and metastatic HCC with sorafenib and SBRT improved PFS and OS compared to those receiving sorafenib alone [16]. The majority of patients in RTOG 1112 (n=131/177; 74%) had macrovascular invasion, which is challenging to successfully treat with other LRT modalities and may represent an opportunity to treat patients with SBRT [16]. A series of three patients by Walburn et al. demonstrated the feasibility of delivering SBRT to isolated nodal recurrences with or without prior OLT [17]. Of those three patients, one demonstrated stable disease in the treated lymph node, and the other two patients demonstrated complete or near-complete responses with no evidence of progression [17]. In our study, Patient 1 presented with oligometastases in the right lung (two sites) and right adrenal gland (one site) that were treated synchronously with the liver graft recurrence. All of these lesions (n=4) maintained 100% LC over the follow-up period, and post-SBRT survival was 25.8 months. These studies suggest that ablative SBRT for limited intra- and extrahepatic metastases may provide a meaningful disease-free survival benefit and should be further evaluated, particularly when combined with the best available ST strategy.

For disseminated (non-oligometastatic) HCC recurrences, the mainstay of treatment remains ST due to the presence of widespread disease and the lack of any benefit of LRT in this setting. There are significant concerns, however, regarding immunotherapy use in the post-transplant setting, as there may be negative interactions with immunosuppressive regimens and a substantial risk for graft rejection [18]. Tyrosine kinase inhibitors (TKIs), such as sorafenib, are the mainstay of treatment for patients with disseminated HCC in the post-transplant setting. However, modest improvements in OS are often countered by drug-related toxicities [4,19]. More recent studies in the post-OLT setting have demonstrated improved tolerability of newer TKIs such as lenvatinib and regorafenib [20]. For non-transplant patients, however, the utilization of anti-PD-1/PD-L1 immune checkpoint blockade (ICB) therapies has substantially improved survival outcomes compared to TKIs in patients with metastatic HCC. As ICB treatments continue to expand into practice, further clinical trials will be needed to determine the safety of such regimens in the post-OLT setting. The synergistic effect of SBRT with immunotherapy is well-documented, as SBRT can promote the expansion of tumor-antigen-specific T cells and increase tumoral MHC-I expression, leading to more effective T cell-mediated killing. Additional prospective clinical studies are warranted to test whether the same benefit afforded in primary, advanced HCC may safely be extended to the setting of post-OLT recurrences.

## Conclusions

In the current report, we demonstrated the successful use of SBRT for the treatment of intrahepatic and extrahepatic HCC recurrences following OLT. SBRT demonstrated excellent local control with no apparent toxicities in this case series. Additional prospective studies are needed to evaluate the potential quantitative survival benefit of SBRT in the post-OLT setting. Furthermore, systemic and out-of-field progression underscores the need for continued surveillance in this population and the need for multimodal treatment strategies. As the utilization of SBRT expands beyond confined intrahepatic disease, further studies investigating the efficacy and safety of SBRT in the setting of extrahepatic disease and in combination with ST are warranted.
